# Comparative and phylogenetic analysis of complete chloroplast genomes from seven *Neocinnamomum* taxa (Lauraceae)

**DOI:** 10.3389/fpls.2023.1205051

**Published:** 2023-07-07

**Authors:** Zhengying Cao, Linyi Yang, Yaxuan Xin, Wenbin Xu, Qishao Li, Haorong Zhang, Yuxiang Tu, Yu Song, Peiyao Xin

**Affiliations:** ^1^ Southwest Research Center for Landscape Architecture Engineering, National Forestry and Grassland Administration, Southwest Forestry University, Kunming, China; ^2^ Key Laboratory of Forest Resources Conservation and Utilization in the Southwest Mountains of China Ministry of Education, Southwest Forestry University, Kunming, China; ^3^ Yunnan Forestry Vocational and Technical College, Kunming, Yunnan, China; ^4^ Wuhan Botanical Garden, Chinese Academy of Sciences, Wuhan, Hubei, China; ^5^ Key Laboratory of Ecology of Rare and Endangered Species and Environmental Protection (Ministry of Education) & Guangxi Key Laboratory of Landscape Resources Conservation and Sustainable Utilization in Lijiang River Basin, Guangxi Normal University, Guilin, Guangxi, China

**Keywords:** *Neocinnamomum*, chloroplast genome, genome comparison, sequence characteristic, phylogenetic analysis

## Abstract

The genus *Neocinnamomum* is considered to be one of the most enigmatic groups in Lauraceae, mainly distributed in tropical and subtropical regions of Southeast Asia. The genus contains valuable oilseed and medicinal tree species. However, there are few studies on the genus *Neocinnamomum* at present, and its interspecific relationship is still unclear. In order to explore the genetic structure and evolutionary characteristics of the *Neocinnamomum* chloroplast genome and to resolve the species relationships within the genus, comparative genomic and phylogenetic analyses were performed on the whole chloroplast genome sequences of 51 samples representing seven *Neocinnamomum* taxa. The whole *Neocinnamomum* chloroplast genome size ranged from 150,753-150,956 bp, with a GC content of 38.8%-38.9%. A total of 128 genes were annotated within the *Neocinnamomum* chloroplast genome, including 84 protein coding genes, 8 rRNA genes, and 36 tRNA genes. Between 71-82 SSRs were detected, among which A/T base repeats were the most common. The chloroplast genome contained a total of 31 preferred codons. Three highly variable regions, *trnN*-*GUU*-*ndhF*, *petA*-*psbJ*, and *ccsA*-*ndhD*, were identified with Pi values > 0.004. Based on the whole chloroplast genome phylogenetic tree, the phylogenetic relationships among the seven *Neocinnamomum* taxa were determined. *N. delavayi* and *N. fargesii* were the most closely related species, and *N. lecomtei* was identified as the most basal taxon. In this study, the characteristics and sequence variation of the chloroplast genomes of seven *Neocinnamomum* taxa were revealed, and the genetic relationship among the species was clarified. The results of this study will provide a reference for subsequent molecular marker development and phylogenetic research of *Neocinnamomum*.

## Introduction

*Neocinnamomum* (Neocinnamomeae, Lauraceae) is a genus of evergreen shrubs and small trees distributed across south-central, western, and northwestern China, as well as Nepal, Myanmar, and Vietnam, among other areas (Flora of China, 1982). To date, there are 7 known species of *Neocinnamomum* worldwide. Five of these species (*N. caudatum*, *N. fargesii*, *N. lecomtei*, *N. mekongense*, and *N. delavayi*) are located in China, and are primarily distributed across Hainan, Guangxi, Yunnan, and Sichuan, as well as Tibet (Flora of China, 1982). In 2017, Xu et al. (2017) discovered a variant of *N. caudatum* (*N. caudatum* var. *macrocarpum*) in Baise, Guangxi, China. Outside of China, *N. atjehense* and *N. parvifolium* (revised as *N. delavayi*) are also recorded in the world flora (WFO, 2023). *Neocinnamomum* includes important oil-bearing tree species as well as valuable medicinal resources. In China, *N. caudatum* is known as “Baigui” and *N. delavayi* is known as “Sangujin” (Wu, 1983). The bark and leaves of these species have a long history of use in traditional Chinese medicine (TCM) for dispelling wind and cold, promoting blood circulation, and relieving blood stasis (Jiangsu New Medical College, 1977). In addition, the seeds of *Neocinnamomum* species are rich in fatty acids (Gan et al., 2018). For example, the seeds of *N. caudatum* contain approximately 54% oil (dry weight), and the seeds of *N. delavayi* contain approximately 57% oil. These oils have the potential to be developed and promoted as raw materials for biodiesel production (Wang et al., 2013), which is important for sustainable energy production. Genomic research on the genus *Neocinnamomum* currently lags far behind that of other oil plants. In addition, the degree of exploitation and utilization of wild *Neocinnamomum* resources is also extremely low.

The genus *Neocinnamomum* was originally established by Liu in 1934, and was distinguished from other genera of Lauraceae by the presence of four-locular anthers with collateral pollen sacs (Liu, 1934). However, this feature appears to only exist in *N. delavayi* and a few individuals of *N. caudatum*. Because of this, Kostermans (Kostermans, 1974) suggested that these anther characteristics were insufficient to distinguish the genus *Neocinnamomum*. Instead, he suggested that the presence of compound cymes; shallow, fleshy fruiting receptacles; persistently enlarged tepals; and dichotomous leaves were better distinguishing characteristics. Despite these traits, Kostermans did not deny the morphological similarity between *Neocinnamomum* and *Cinnamomum*. Traditional classification systems have long considered the two genera to be closely related. However, more recent molecular phylogenetic studies have called this relationship into question, obscuring the phylogenetic position of the genus *Neocinnamomum* within Lauraceae. The development of molecular systematics techniques has allowed researchers to utilize chloroplast gene fragments, whole chloroplast genomes, and nrDNA to study *Neocinnamomum* phylogenetics (Chanderbali et al., 2001; Rohwer and Rudolph, 2005; Wang et al., 2010; Song et al., 2020). In contrast to the traditional understanding that *Neocinnamomum* and *Cinnamomum* are closely related, these recent studies indicate that *Neocinnamomum* is a monophyletic group with *Cassytha* and *Caryodaphnopsis* as its closest relatives.

Although the monophyly of the genus *Neocinnamomum* has been confirmed, the relationships between species within the genus are less clear. Kostermans (1974) divided the genus *Neocinnamomum* into six species, four of which are easily distinguished. However, *N. mekongense* and *N. delavayi* are extremely similar morphologically, and can be distinguished only based on the presence of pubescent twigs. Recently, Wang et al. (2010) used three molecular fragments of *psbA-trnH*, the *trnK* cpDNA region, and ITS nrDNA to draw a phylogenetic tree of the genus *Neocinnamomum* and found that *N. mekongense* and *N. delavayi* were located on the same branch. Despite this, molecular fragment analysis has not satisfactorily resolved the relationship between the two species. Compare with the use of chloroplast (cp) gene fragments, the whole cp genome can provide much more robust variation information (Wariss et al., 2017). For example, Ren et al. (2019) constructed a maximum likelihood (ML) phylogenetic tree using the whole chloroplast genomes of *N. lecomtei*, *N. mekongense*, and *N. delavayi*. This highly-supported analysis indicated that *N. lecomtei* and *N. mekongense* were located on different branches. Unfortunately, only 5 samples from 3 species of *Neocinnamomum* were used for the study, limiting the power of the results.

Chloroplasts are the central hub for photosynthetic and certain other metabolic reactions in plants (Tao et al., 2017). Like mitochondria, cp are maternally inherited semi-autonomously and contain a relatively independent genetic system. In addition, cp have been central drivers of evolutionary processes (Neuhaus and Emes, 2000; Xing and Liu, 2008). As sequencing technology has developed, the publication of an ever-increasing number of plant cp genomes has given us a deeper understanding of the structure and variation of cp genomes. For the vast majority of angiosperms, the cp genome is characterized by a closed, double-stranded circular structure, and consists of two inverted repeat (IRs) regions, a large single-copy (LSC) region, and a small single-copy (SSC) region (Jansen et al., 2011). The structural stability of the cp genome is largely maintained by the conserved IR region, which is characterized by a base substitution rate of only 1/4 of that of the SC region (Drouin et al., 2008). The cp genome offers several advantages over the nuclear and mitochondrial genomes, including structural conservation, low molecular weight, simple structure, genetic stability, and moderate evolution rate (slower than the nuclear genome but higher than the mitochondrial genome) (Goremykin et al., 2003). Owing to these unique characteristics, cp genomes are widely used to explore the phylogeny and genetic relationship among plant clades (Yang et al., 2018; Cao et al., 2022; Lan et al., 2022; Wang et al., 2023; Xin et al., 2023). The cp genome is particularly advantageous for studies of species identification, molecular geography, and speciation processes (Xu et al., 2001).

To date, only a few *Neocinnamomum* genomes have been sequenced, and detailed cp genomic comparisons and phylogenetic analyses are lacking. In order to further clarify the phylogenetic relationships among species of the genus *Neocinnamomum*, and to obtain useful genetic resources, we sequenced and assembled the cp genomes of 50 samples representing 7 *Neocinnamomum* taxa. Included in the analysis was one unique taxa recently collected from Wenshan, Yunnan, China, which shares some traits with *N. complanifructum* (merged into *N. lecomtei*). Based on 51 cp genome sequences (including 50 sequencing sequences and one sequence from NCBI), we analyzed the cp genome structure, gene content, codon usage frequencies, simple sequence repeats (SSRs), highly variable regions, and IR expansion and contraction. Finally, we reconstructed the phylogenetic relationships of 7 *Neocinnamomum* taxa. These results will be of great significance for studies of the population genetics, species identification, and conservation biology of the genus *Neocinnamomum*.

## Materials and methods

### Plant material sampling

Fifty samples of either fresh leaves or silica gel-dried material representing 7 *Neocinnamomum* species were collected, including 5 samples of *N. delavayi*, 6 samples of *N. mekongense*, 4 samples of *N. fargesii*, 2 samples of *N. caudatum* var. *macrocarpum*, 8 samples of *N. lecomtei*, 23 samples of *N. caudatum*, and 2 samples of *N. sp* (**Supplementary Table S1**). Plant samples were primarily collected in the Yunnan, Sichuan, Guangxi, and Hainan provinces of China, at an altitude of 1100-2300 m. Only fresh, tender, healthy leaves with no visible pest or disease damage were collected. Fresh samples were dried in self-sealed bags of silica gel prior to DNA extraction and sequencing. At the same time, the cp genome sequence data of *N. caudatum* (RL01) was downloaded from NCBI database for subsequent analysis.

### DNA extraction and sequencing

Total genomic DNA was extracted from fresh and silica gel-dried leaves using a modified cetyltrimethylammonium bromide (CTAB) method (Doyle and Doyle, 1987). DNA integrity was evaluated using 1% agarose gel electrophoresis. DNA purity and concentration were evaluated by Nanodrop. Using qualified DNA samples, 150 bp double-end libraries were constructed and sequenced on the Illumina sequencing platform at Novogene Bioinformatics Technology Co. Ltd (Beijing, China). More than 4.0 Gb of reads was generated per sample.

### Genome assembly and annotation and codon preference analysis

After the sequencing data were filtered and screened, the cp genome was automatically assembled using the GetOrganelle v1.7.1 (Jin et al., 2020). Bandage v0.8.1 (Wick et al., 2015) was used to visualize the assembly, remove redundant contigs, and edit the sequences into loops. The *N. mekongense* whole cp genome sequence (GenBank accession number NC_039718) was used as a reference, automatically annotated using CPGAVAS2 (http://47.96.249.172:16019/analyzer/home) (Liu et al., 2012), and adjusted using Geneious v9.1.7 (Kearse et al., 2012). The circular cp genome diagram was created using the OGDRAW online tool (https://chlorobox.mpimp-golm.mpg.de/OGDraw.html) (Lohse et al., 2013). The final assembled and annotated cp genome sequences were uploaded to the NCBI database to obtain GenBank accession numbers.

Eleven individuals of *Neocinnamomum* plants were selected for analysis (one sample was randomly selected from each taxon, and RL01 *N. caudatum*, 7751 N*. caudatum*, 6068 N*. delavayi* and 7683 N*. mekongense*).The relative synonymous codon usage (RSCU) of 11 cp genomes was statistically analyzed using CodonW1.4.2 software (https://galaxy.pasteur.fr/?form=codonw) (Liu and Xiu, 2005). Frequent codon usage (i.e., codon usage preference) is indicated when RSCU >1, while RSCU = 1 indicates no usage preference.

### Simple sequence repeat analysis

The SSRs present in 11 cp genomes were detected by MISA (https://webblast.ipk-gatersleben.de/misa/) (Beier et al., 2017), using the following parameters: the threshold values for the number mononucleotide, dinucleotide, trinucleotide, tetranucleotide, pentanucleotide, and hexanucleotide repeats were set to 10, 5, 4, 3, 3, and 3, respectively (Wu et al., 2021).

### Comparative chloroplast genome analysis

By examining the genes bordering IR regions, we can analyze IR expansion and contraction within the cp genomes of *Neocinnamomum* species. We manually examined the IR junctions of 11 cp genomes in Geneious software. To identify differences among the 11 cp genomes of the *Neocinnamomum* taxa, a comparative analysis of the full-length cp genome sequences was performed using mVISTA (http://genome.lbl.gov/vista/mvista/submit.shtml) (Frazer et al., 2004), with the cp genome of *N. delavayi* (6068) used as a reference. MAFFT v7.455 software (Katoh and Standley, 2013) was used to align the 51 cp genomes. The nucleotide polymorphism (Pi) between genomes was calculated using DnaSP v6.12.03 (Rozas et al., 2017), with a window length of 600 and a step size of 200 (Shen et al., 2022). The high-variance regions were screened by combining the mVISTA and Pi results.

### Phylogenetic analysis

Phylogenetic relationships were reconstructed based on the 51 cp genomes, using *Caryodaphnopsis henryi*, *C. tonkinensis* and *C. malipoensis* from NCBI database as outgroup. Both the ML and Bayesian Inference (BI) methods were used to construct the phylogenetic tree. Each of the complete cp genomes were aligned using MAFFT v7.455 (Katoh and Standley, 2013), and then manually edited using BioEdit v 7.2.5 (Hall, 1999). IQ-TREE v1.6.7 (Nguyen et al., 2015) was used to construct the ML phylogenetic tree, and the “GTR+F+R4” model was used for nucleic acid substitution. The support of each branch was verified by bootstrapping, with 1,000 iterations. BI analyses were performed using the Mrbayes v3.2.6 module of the CIPRES website (http://www.phylo.org/) (Huelsenbeck and Ronquist, 2001). Briefly, the processed data was uploaded to the CIPRES platform and jModeltest v2.1.10 (Posada, 2008) was used to determine the best nucleotide substitution model for phylogenetic reconstruction. Then, the Markov chain Monte Carlo (MCMC) algorithm was run for 1,000,000 generations. The results were sampled every 100 generations, and the first 25% of the generated trees were discarded. The “TPM1uf+I+G” model (freqA=0.3027, freqC=0.1972, freqG=0.1894, freqT=0.3107, R(a)[AC]=1.0000, R(b)[AG]=2.8265, R(c)[AT]=0.3346, R(d)[CG]=0.3346, R(e)[CT]=2.8265, R(f)[GT]=1.000, p-inv=0.6960, gamma shape=1.0440) was used to construct the BI phylogenetic tree. The phylogenetic trees were visualized and adjusted using FigTree v1.4.3 (Dong et al., 2022b).

## Results

### Features of the chloroplast genome

**Figure 1:**
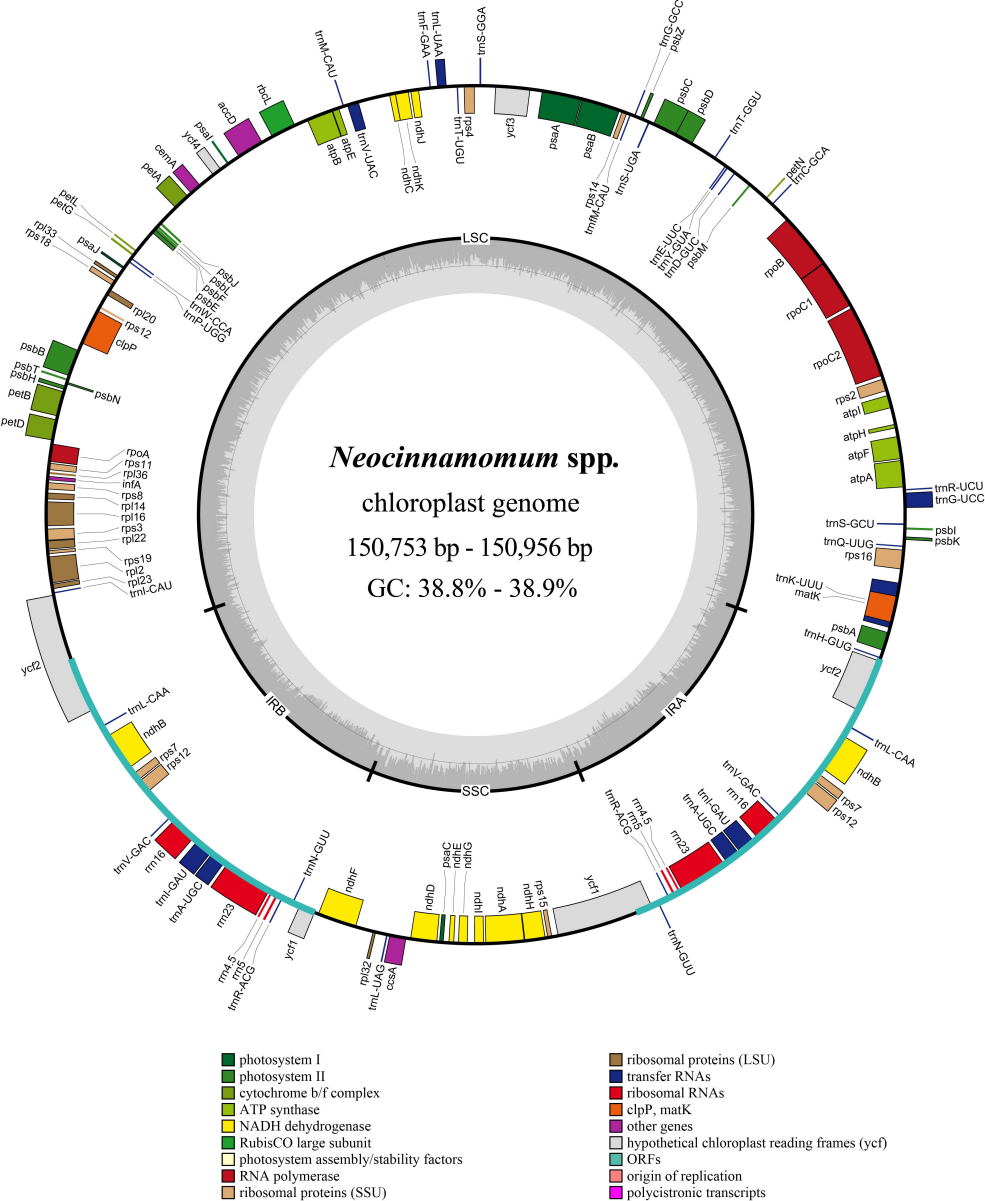
Circular map of the *Neocinnamomum* chloroplast genome. Genes belonging to different functional groups are color-coded. In the inner circle, the GC and AT content are denoted by the dark gray and light gray, respectively.

The structure of the *Neocinnamomum* cp genome is a typical circular double-stranded tetrad (**Figure 1**). The cp genome size of 51 *Neocinnamomum* plant samples ranged from 150,753 to 150,956 bp (**Supplementary Table S2**). The GC content was between 38.8%-38.9%, with *N. lecomtei* and *N. mekongense* having a higher GC content than the other studied taxa. We observed some variation in the length and GC content of the LSC, SSC, and IR regions of the cp genomes. The LSC region ranged in length between 91,850-92,006 bp, and had a GC content of 37.4%-37.5%. The SSC region ranged in length between 18,096-18,457 bp, and had a GC content of 33.2%-33.4%. The IR region ranged in length between 20,257-20,425 bp, and had a GC content of 44.5%-44.6%. Overall, the GC content of the IR region was significantly higher than in the SSC and LSC regions.

A total of 128 genes were annotated within the *Neocinnamomum* cp genome, including 84 protein coding genes, 8 rRNA genes, and 36 tRNA genes (**Table 1**). The coding genes were primarily composed of self-replication genes, photosynthesis genes, ycf genes, and “other” genes. Two copies each of 15 genes were located in the IR region, including 5 protein coding genes (*ndhB*, *rps7*, *rps12*, *ycf1*, and *ycf2*), 4 rRNA genes (*rrn4.5*, *rrn5*, *rrn16*, and *rrn23*) and 6 tRNA genes (*trnA*-*UGC*, *trnI*-*CAU*, *trnL*-*CAA*, *trnN*-*GUU*, *trnR*-*ACG*, and *trnV*-*GAC*). In addition, 15 genes (9 protein coding genes and 6 tRNA genes) contained one intron and 3 genes (*rps12*, *clpP*, ycf3) contained two introns.

**Table 1:** Genes present in the *Neocinnamomum* chloroplast genome.

Category	Group of Genes	Genes Names
Photosynthesis gene	Photosystems I	*psaA*, *psaB*, *psaC*, *psaJ*, *psaI*
	Photosystems II	*psbA*, *psbB*, *psbC*, *psbD*, *psbE*, *psbF*, *psbH*, *psbI*, *psbJ*, *psbK*, *psbL*, *psbM*, *psbN*, *psbT*, *psbZ*
	Cytochrome b/f complex	*petA*, *petB* ^A^, *petD* ^A^, *petG*, *petL*, *petN*
	ATP synthase	*atpA*, *atpB*, *atpE*, *atpF* ^A^, *atpH*, *atpI*
	NADH dehydrogenase	*ndhA* ^A^, *ndhB* ^A,C^, *ndhC*, *ndhD*, *ndhE*, *ndhF*, *ndhG*, *ndhH*, *ndhI*, *ndhJ*, *ndhK*
	Rubisco Large subunit	*rbcL*
Self-replication gene	RNA polymerase	*rpoA*, *rpoB*, *rpoC1* ^A^, *rpoC2*
	Ribosomal proteins (SSU)	*rps2*, *rps3*, *rps4*, *rps7* ^C^, *rps8*, *rps11*,*rps12* ^B,C^, *rps14*, *rps15*, *rps16* ^A^, *rps18*, *rps19*
	Ribosomal proteins (LSU)	*rpl2* ^A^, *rpl14*, *rpl16* ^A^, *rpl20*, *rpl22*, *rpl23*, *rpl32*, *rpl33*, *rpl36*
	Transfer RNAs	*trnA-UGC* ^A,C^, *trnC-GCA*, *trnD-GUC*, *trnE-UUC*, *trnF-GAA*, *trnfM-CAU*, *trnG-GCC*, *trnG-UCC* ^A^, *trnH-GUG*, *trnI-CAU ^C^*, *trnI-GAU* ^A^, *trnK-UUU* ^A^, *trnL-CAA* ^C^, *trnL-UAA* ^A^, *trnL-UAG*, *trnM-CAU*, *trnN-GUU* ^C^, *trnP-UGG*, *trnQ-UUG*, *trnR-ACG* ^C^, *trnR-UCU*, *trnS-GCU*, *trnS-GGA*, *trnS-UGA*, *trnT-GGU*, *trnT-UGU*, *trnV-GAC* ^C^, *trnV-UAC* ^A^, *trnW-CCA*, *trnY-GUA*
	Ribosomal RNAs	*rrn4.5* ^C^, *rrn5* ^C^, *rrn16* ^C^, *rrn23* ^C^
Other genes	Maturase	*matK*
	Envelop membrane protein	*cemA*
	Subunit of acetyl-CoA-carboxylase	*accD*
	Translational initiation factor	*infA*
	C-type cytochrome synthesis	*ccsA*
	Proteolysis	*clpP* ^B^
Functions unknown	Hypothetical chloroplast reading frames(ycf)	*ycf1* ^C^, *ycf2* ^C^, *ycf3* ^B^, *ycf4*

A and B indicate one intron and two introns, respectively. C indicates two copies of the gene.

### Codon bias analysis

**Figure 2:**
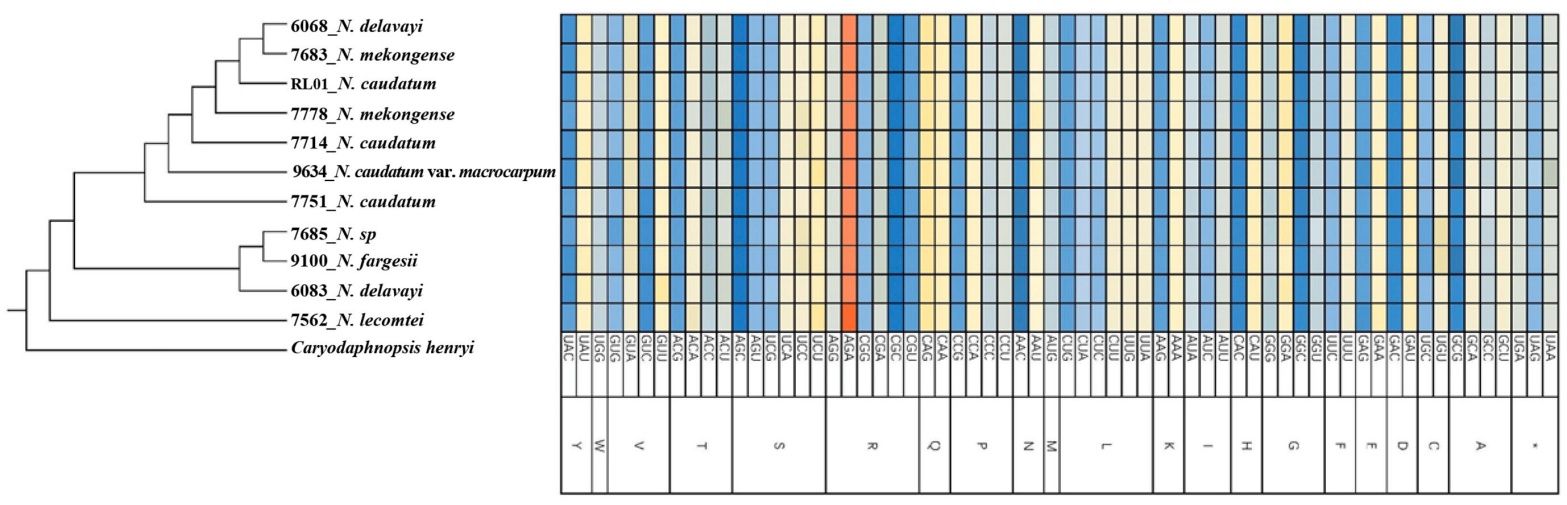
Relative synonymous codon usage (RSCU) of 20 amino acids and the stop codon for 11 chloroplast genomes. 20 uppercase letters such as A, C and D are abbreviations of 20 amino acids, and the symbol "*" is the stop codon.

Using MEGAX64 to determine the codon usage bias of the cp genomes, 64 codons, encoding 20 amino acids, were detected (**Figure 2**). Among these, three codons had RSCU = 1 and 31 codons had RSCU > 1, indicating a preference for these codons within the *Neocinnamomum* cp genome. Among 11 cp genomes of the 7 *Neocinnamomum* taxa, the AGA codon encoding arginine (R) was the most frequently used among the synonymous codons, with RSCU values ranging from 1.82-1.83. Least frequently used was the CGC codon encoding arginine (R), with RSCU values ranging from 0.41-0.42. Among the 20 amino acids, the most common were arginine (R), leucine (L), and serine (S), using 6 codons. The next most common were alanine (A), glycine (G), proline (P), threonine (T), and valine (V), using 4 codons. The least common were tryptophan (W) and methionine (M), using 1 codon. Except for methionine (M) and tryptophan (W), the preferred codons for all amino acids ended in A/U, indicating a preference for codons ending in these bases across the *Neocinnamomum* cp genome.

### Simple sequence repeats analysis

**Figure 3:**
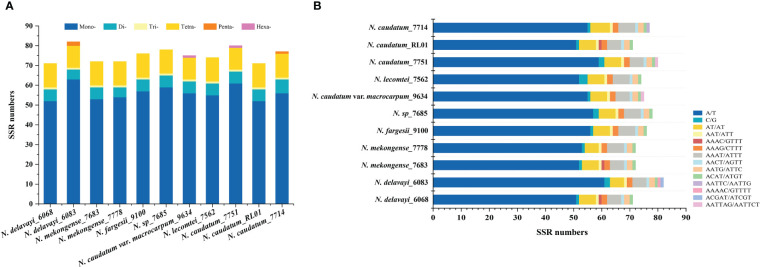
Comparison of SSRs across 11 *Neocinnamomum* cp genomes. **(A)** The type frequency of different SSR types. **(B)** The type frequency of SSR motifs in different repeat class types.

SSRs are widely distributed in chloroplast genomes. We detected between 71-82 SSRs across each of 11 cp genomes representing 7 *Neocinnamomum* taxa, for a total of 828 SSRs. The SSRs ranged from single to hexanucleotide repeats, although not all six types of SSR were present in each taxa (**Figure 3**). Single nucleotide SSRs were the most common (52-63), and were mainly comprised of A/T repeats, with only 1-3 C/G repeats. There were 5-7 dinucleotide SSRs, all of which were AT/AT repeats. Only one trinucleotide SSR, an AAT/ATT repeat, was identified, with only one repeat per sample. There were 11-12 tetranucleotide SSRs, including four repeat types. However, the AAAC/GTTT repeat was present only in *N. delavayi* (6068), *N. mekongense* (7683), and *N. caudatum* (RL01). There were 0-2 pentanucleotide SSRs, including three repeat types (AATTC/AATTG, AAAAC/GTTTT, and ACGAT/ATCGT), which were present only in *N. delavayi* (6083) and *N. caudatum* (7714). The hexanucleotide AATTAG/AATTCT SSR was identified only in *N. caudatum* var. *macrocarpum* and *N. caudatum* (7751), with one repeat per sample.

### Contraction and expansion of the IR regions

**Figure 4:**
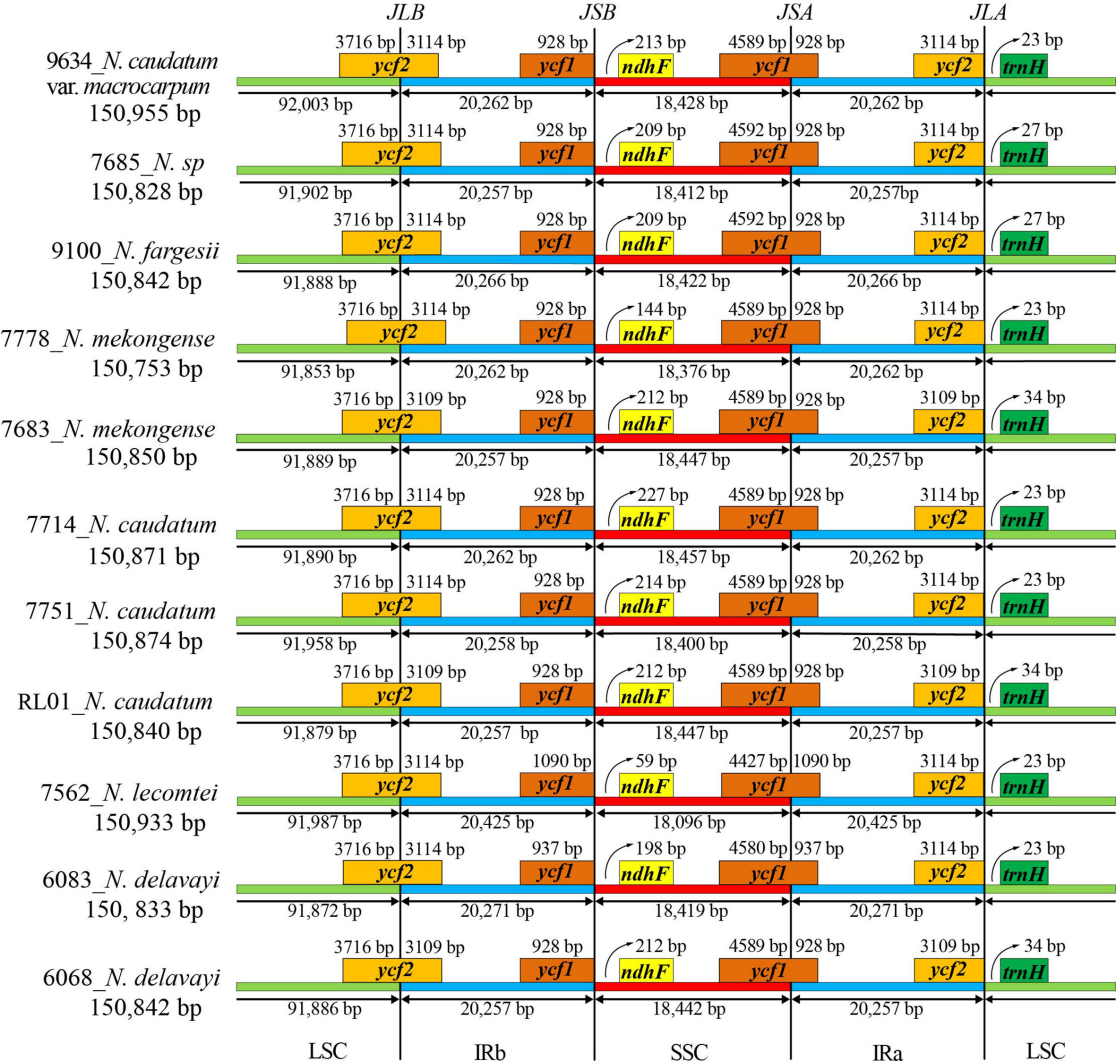
Expansion and contraction of the IR/SC boundary of the *Neocinnamomum* chloroplast genome.

IR junctions analysis of 11 cp genomes representing 7 *Neocinnamomum* taxa indicated the presence of a contracted expansion of the tetrad structure SC/IR boundary (**Figure 4**). The LSC/IRb (JLB), IRb/SSC (JSB), SSC/IRa (JSA), and IRa/LSC (JLA) linkage boundaries were primarily associated with four genes: *ycf1*, *ycf2*, *ndhF*, and *ndhH*. The LSC/IRb (JLB) boundary and the SSC/IRa (JSA) boundary were located within the coding regions of *ycf2* and *ycf1*, respectively. In three samples (7683, 6068, and RL01), the *ycf2* gene expanded 3109 bp into the IRb region, which was 5 bp less than in other samples. The *ycf1* gene is different in both *N. delavayi* and *N. lecomtei* compared to the other taxa, with an additional expansion of 9 bp and 162 bp into the IRa region, respectively. The IRb/SSC (JSB) boundary was located in the noncoding region between *ycf1* and *ndhF*, with *trnF* of *N. lecomtei* closest to the boundary at 59 bp. The IRa/LSC (JLA) boundary was located in the noncoding region between *ycf2* and *ndhH*, with *trnH* of three samples (7683, 6068, and PL01) farthest from the boundary at 34 bp. Interestingly, the IR boundaries of *N. lecomtei* differed significantly from the other taxa. We hypothesize that this taxa either has an increased rate of evolution or diverged earlier. The distribution of genes at the SC/IR boundary was similar for samples 7683, 6068, and PL01, but differed somewhat from other samples of their respective taxa. Collectively, although the SC/IR boundary of the *Neocinnamomum* cp genome was relatively conservative, it also exhibited significant diversity.

### Identification of variability hotspots

**Figure 5:**
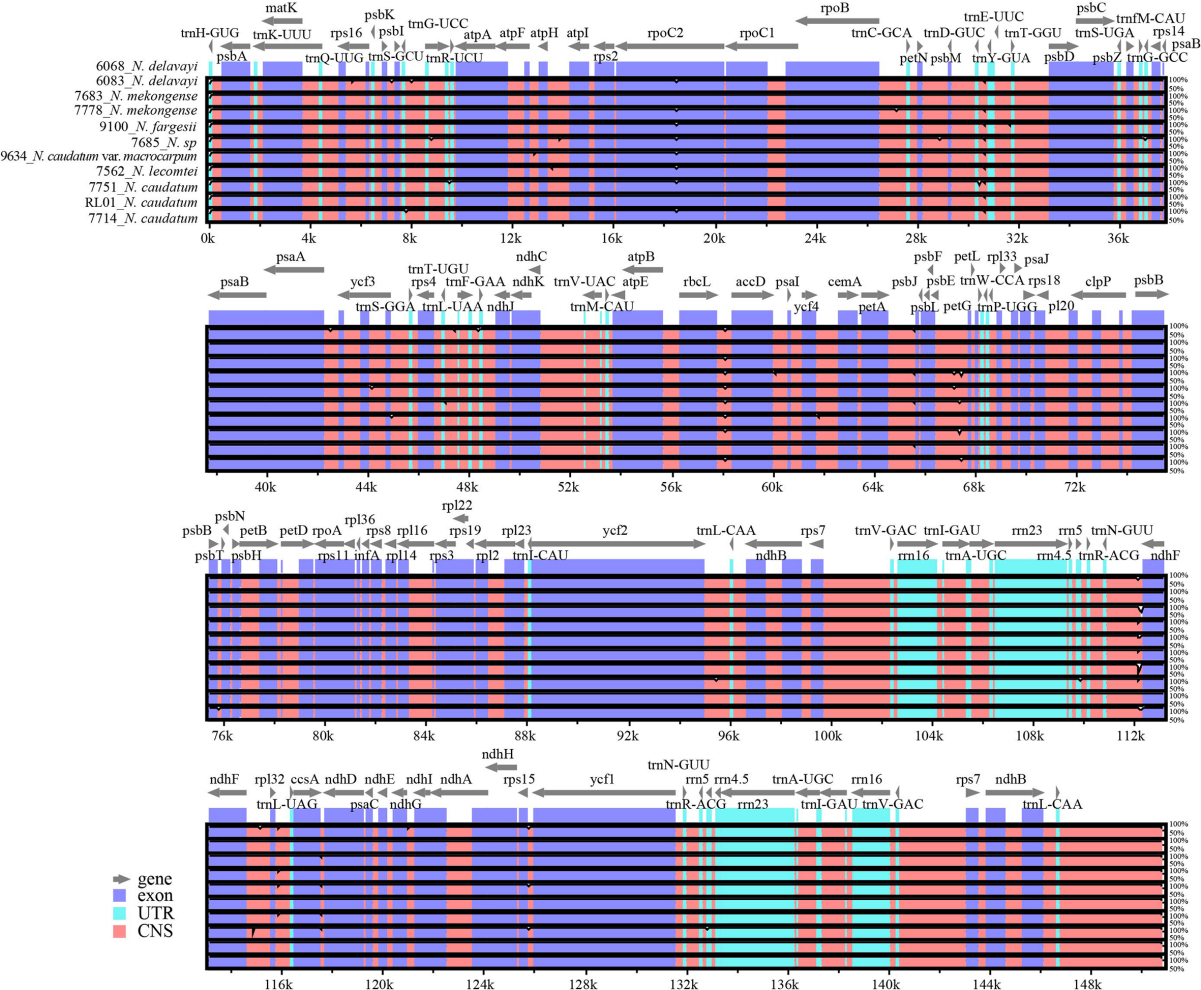
Complete chloroplast genome alignments for seven species of *Neocinnamomum*. mVISTA software was used to perform the alignment, with the *N. delavayi* (6068) chloroplast genome used as the reference sequence. The gray arrows indicate genes and their directions.

Using mVISTA software, the *N. delavayi* (6068) cp genome was used as the reference and compared with the full-length cp genomes of *N. delavayi* (6083), *N. mekongense* (7683 and 7778), *N. fargesii* (9100), *N. caudatum* (7714, 7751 and RL01), *N. caudatum* var. *macrocarpum* (9634), *N. sp* (7685), and *N. lecomtei* (7562) (**Figure 5**). Overall, both gene composition and order was relatively conserved across all 7 *Neocinnamomum* taxa. The IR region was more conserved than the LSC and SSC regions. The coding region was more conserved than the non-coding region, and variants primarily occurred in the spacer regions of adjacent genes, such as *trnN-GUU-ndhF*, *petA-psbJ*, *rbcL-accD*, and *psbE-petL*.

**Figure 6:**
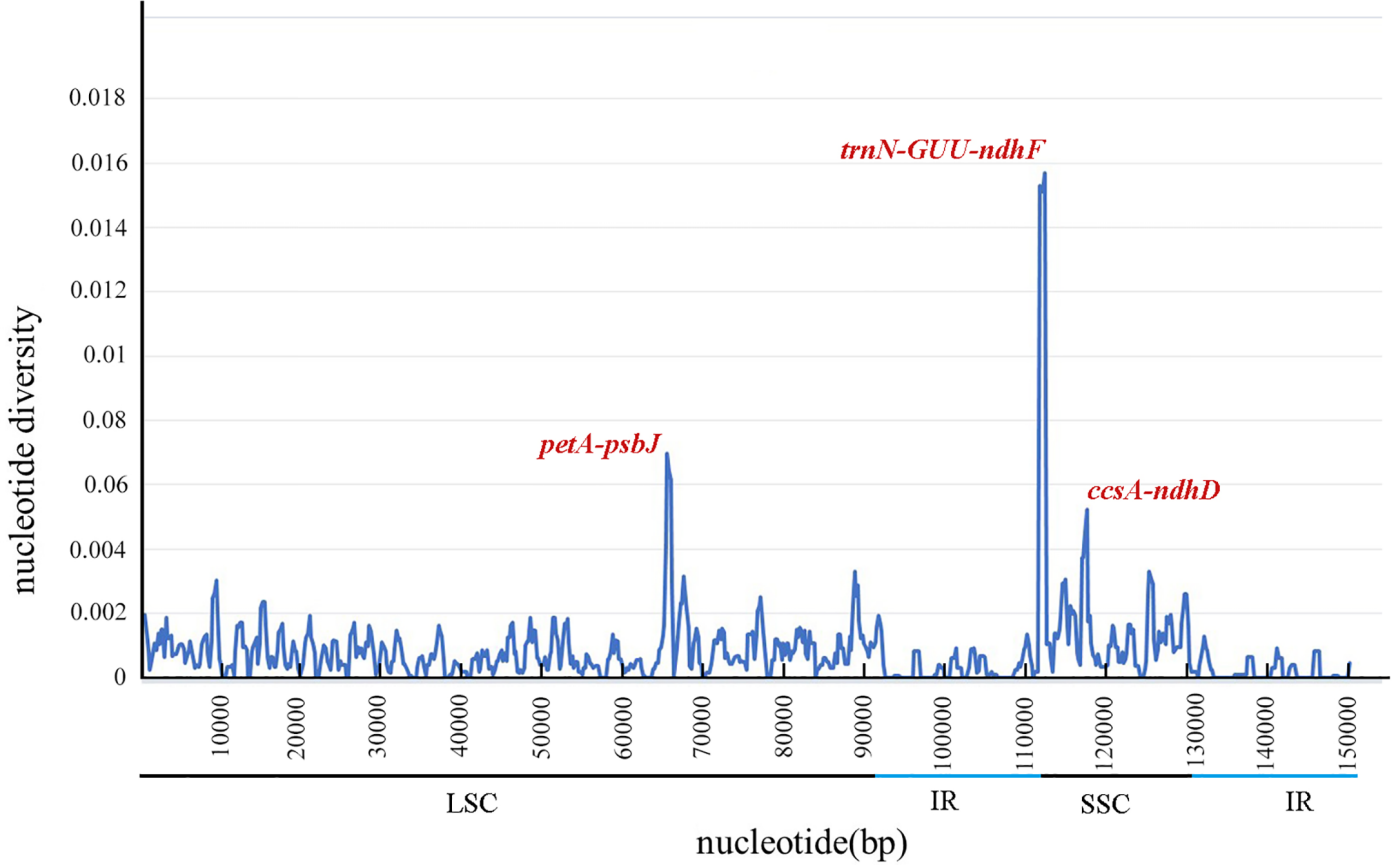
Nucleotide diversity (Pi) in matching areas of *Neocinnamomum* chloroplast genomes.

DnaSP software was used to compare the nucleotide variation values (Pi) between all genes and intergenic regions of the cp genomes. The Pi values of the 51 *Neocinnamomum* cp genomes varied from 0 to 0.01571, with a mean of 0.00098 (**Figure 6**). Although the *Neocinnamomum* cp genome was highly conserved, we identified three divergent hotspot regions (Pi > 0.004): *trnN*-*GUU*-*ndhF*, *petA*-*psbJ*, and *ccsA*-*ndhD*. Among them, *petA*-*psbJ* was located in the LSC region and had a Pi value of 0.00696. Both *trnN*-*GUU*-*ndhF* and *ccsA*-*ndhD* were located in the SSC region and had Pi values of 0.01571 and 0.00522, respectively.

### Phylogenetic analysis

**Figure 7:**
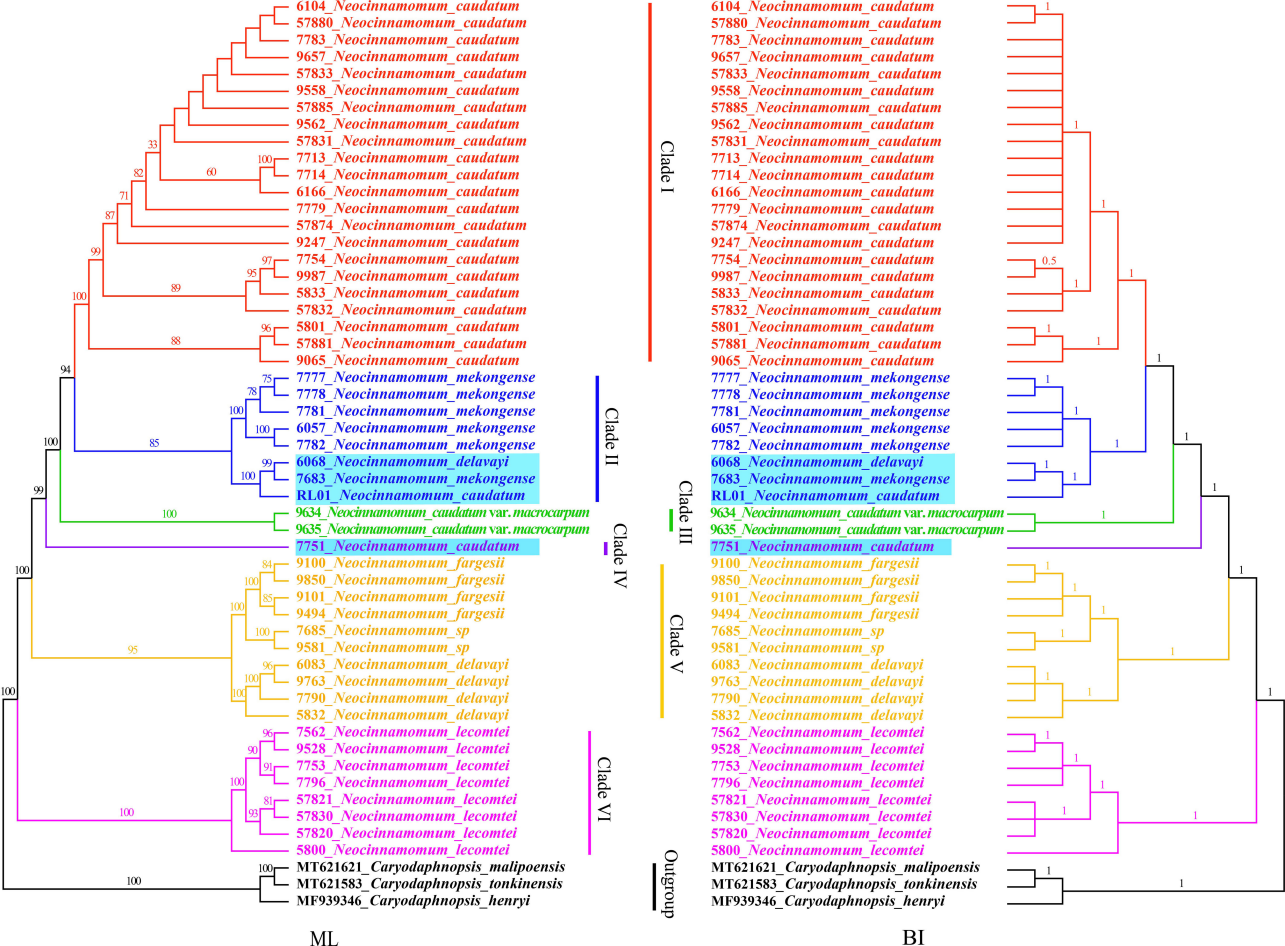
Phylogenetic tree constructed with *Neocinnamomum* chloroplast genomes.

The phylogenetic relationships among 7 taxa of *Neocinnamomum* were reconstructed based on the cp genomes, with *C. henryi*, *C. tonkinensis* and *C. malipoensis* used as outgroup (**Figure 7**). In the ML phylogenetic tree, 40.91% of the branches had 100% support and 93.18% of the branches had ≥ 75% support. In the BI phylogenetic tree, only a small branch had a support rate of 0.5, and the other branches had a support of 1. The topology of the phylogenetic tree constructed based on both ML and BI methods was nearly identical. Overall, the 51 samples representing 7 *Neocinnamomum* taxa were divided into six branches. Clade I included only *N. caudatum*. Clade II included *N. mekongense*, as well as *N. delavayi* (6068) and *N. caudatum* (RL01). Clade III included only *N. caudatum* var. *macrocarpum*. Clade IV included only *N. caudatum* (7751). Clade V included *N. delavayi*, *N. fargesii*, and *N.* sp. Clade VI included only *N. lecomtei*, which was first differentiated as a basal taxon.

## Discussion

The structure, size, and gene content of the cp genomes of the 7 *Neocinnamomum* taxa were relatively conserved. The 51 *Neocinnamomum* cp genomes exhibited a typical tetrad structure, with a length between 150,753 bp-150,956 bp and a GC content of 38.8%-38.9%. Notably, the *Neocinnamomum* chloroplast genome is smaller than that of most other Lauraceae genera (except for *Cassytha*). Song et al. (2017) found that the cp genomes of the core Lauraceae group ranged from 150,749bp to 152,739bp in length, whereas the cp genomes of the basal Lauraceae group ranged from 157,577bp to 158,530bp in length. Their analysis indicated that the core Lauraceae group lost *trnI-CAU*, *rpl23*, *rpl2*, and a fragment of *ycf2*, as well as the intergenic regions of these genes in IRb region. To a great extent, the loss of fragments in the IR region will lead to plastid contraction, which may account for the smaller *Neocinnamomum* cp genome. A total of 128 genes were annotated in the *Neocinnamomum* cp genome, including 84 protein-coding genes, 8 rRNA genes, and 36 tRNA genes. After excluding duplicates, a total of 113 unique genes were identified. These results are consistent with previous studies on *Neocinnamomum* cp genomes (Song et al., 2017; Tian, 2021). Overall, the cp genomes were very similar between all 7 taxa of *Neocinnamomum*, with no significant differences in either gene sequence or gene content.

SSR markers are widely employed in plant germplasm research because they are rich in polymorphism, strongly co-dominant, highly reproducible, stable, and reliable (Echt et al., 1996). We detected a total of 828 SSRs across 11 *Neocinnamomum* cp genomes, with 71-82 detected per sample. Single nucleotide repeats accounted for 74.64% of all SSRs, followed by tetranucleotide repeats (15.58%). These results differ from studies of the Lauraceae genera *Litsea* (Liu et al., 2021) and *Ocotea* (Trofimov et al., 2022), the cp genomes of which contain more dinucleotide repeats than tetranucleotide repeats. According to the principle of base complementarity, the 6 repeat types include 14 repeat motifs, among which A/T accounted for 72.71% of all SSRs, followed by AT/AT and AAAT/ATTT, which accounted for 7.85% and 7.25% of all SSRs, respectively. These results indicate that A/T are the preferred bases across the cp genomes of *Neocinnamomum* taxa. Studies suggest that energy consumption affects base preference. Because the nitrogen content of A/T bases is lower than that of G/C bases, A/T enrichment can make base mutations consume less energy (Niu et al., 2017). In addition to affecting the SSR types and codon bias, base bias can also affect the stability of the four partitions of the cp genome (Mukhopadhyay et al., 2007). To date, there are few published studies of SSR markers in the *Neocinnamomum* cp genome. The identification of SSRs in the *Neocinnamomum* cp genome can provide a reference for the development of molecular markers and the analysis of genetic variation within the genus *Neocinnamomum*.

During cp genome evolution, the boundaries of the IR region may undergo contraction and expansion. This process is central to cp genome evolution and is the primary driver of cp genome diversity among species (Park et al., 2018). Song et al. (2017) detected a double intact copy of the *ycf2* gene, as well as one intact copy and one fragment of *ycf1*, in the plastids of basal Lauraceae group. However, only one intact copy and one fragment of both *ycf2* and *ycf1* were detected in the plastids of *Neocinnamomum*. Consistent with Song’s results, we identified one complete copy and one fragment of *ycf1* and *ycf2* located at the boundary between the IR region and the SC region, respectively. The *ycf1* fragment at the IRb/SSC boundary and the *ycf2* fragment at the IRa/LSC boundary were considered pseudogenes. Zhao et al. (2018) found that variation in the lengths of *ycf1*, *ycf2*, *ψycf1*, and *ndhF*-*ψycf1* drives the contraction and expansion of the IR region in the *Lindera* cp genome. This is similar to *Neocinnamomum*, wherein the contraction and expansion of the IR region was driven by variation in the lengths *ycf1*, *ycf2*, *ndhF*-ψycf1, and *trnH*-*ψycf2*.

Although the cp genome is relatively conserved and exhibits a slow rate of evolution, it also contains several mutation sites such as t*rnH*-*psbA* and *trnQ*-*rps16*, among other fragments (Nithaniyal and Parani, 2016; Linh et al., 2022). We performed a comparative analysis of the cp genomes of 7 *Neocinnamomum* taxa by combining the use of the DnaSP 6 software with the mVISTA online program. Three hypervariable regions, *trnN*-*GUU*-*ndhF*, *petA*-*psbJ*, and *ccsA*-*ndhD*, were identified. These hypervariable regions can be used as candidate molecular markers for the development of specific DNA barcodes to aid in the taxonomic identification of *Neocinnamomum* samples. Among these, the highly-variable *petA*-*psbJ* was previously identified as a variation hotspot in the Lauraceae species *Litsea glutinosa*, *Persea americana*, and *Machilus chuanchienensis* (Hinsinger et al., 2017; Song et al., 2016; Bai et al., 2022). The variability of the IR region was significantly lower than that of the SSC and LSC regions, and the variability of the coding regions was much lower than that of the noncoding regions, in the *Neocinnamomum* cp genome. This result is consistent with studies of cp genomes in other higher plants, including *Hernandia nymphaeifolia* (Li et al., 2020) and *Saposhnikovia divaricata* (Yi et al., 2022). These results also suggest that the noncoding regions evolve faster than the coding regions in the genus *Neocinnamomum*. Therefore, priority should be given to noncoding regions when screening DNA barcodes for *Neocinnamomum* species.

*Neocinnamomum* has been confirmed as a monophyletic by several studies. However, the phylogenetic relationships between species within the genus remain controversial. *N. delavayi* and *N. mekongense* are virtually indistinguishable in terms of morphological and molecular characteristics, and *N. mekongense* was once considered to be a variety of *N. delavayi* (Handel-Mazzetti, 1925). Wang et al. (2010), based on the phylogenetic study of *psbA*-*trnH*, *trnK*, and ITS, reported that *N. delavayi* and *N. mekongense* are closely related sister taxa. Li et al. (2016) used four species of *Neocinnamomum* in a phylogenetic study of *Caryodaphnopsis* based on RPB2, LEAFY, and ITS sequences, and the results supported the sister relationship between *N. delavayi* and *N. mekongense*. However, our phylogenetic analysis yielded different results. In our whole cp genome phylogenetic tree, *N. delavayi* was most closely related to and formed a sister group with *N. fargesii* and *N. sp*, while it was distant from *N. mekongense*. We speculate that the selection and combination of different gene fragments led to divergent phylogenetic trees. In addition, differences in biparental and maternal inheritance could have caused the divergent results of the ITS and cpDNA evolutionary trees (Wu, 2018). Of course, sampling bias may also lead to phylogenetic errors, and increasing the sampling size can effectively improve the overall phylogenetic accuracy (Murphy et al., 2001; Zwickl and Hillis, 2002; Dong et al., 2022a). Although the results are different, it has been recognized that the cp genome can well analyze the phylogeny of Lauraceae, and the whole cp genome is more effective than the fragment (Tian et al., 2021). *N. sp*, a special taxa recently collected from Wenshan, Yunnan, China, is morphologically similar to *N. complanifructum*, which has been merged into *N. lecomtei*. Our results indicated that *N. sp* is a sister taxon of *N. fargesii*. In addition, nesting was observed for 4 samples, including *N. caudatum* (RL01), *N. caudatum* (7751), *N. delavayi* (6068), and *N. mekongense* (7683), which may have been due to partial gene exchange between closely distributed species (Wu et al., 2022). The geographic distributions of *N. delavayi*, *N. mekongense*, and *N. caudatum* are similar, and often overlap, suggesting that hybridization may have occurred among these species. However, whether these four samples represent hybrids will require further morphological and molecular studies.

## Conclusions

In this study, we sequenced and assembled 50 cp genomes representing 7 *Neocinnamomum* taxa, and obtained the cp genome data of *N. caudatum* (RL01) from NCBI. A total of 51 cp genomes were analyzed. Similar to most angiosperms, the *Neocinnamomum* cp genome exhibited a relatively conserved gene structure and gene content. Sequence variations were primarily concentrated in the LSC and SSC regions. Three hotspot regions and 71-82 SSRs were identified, which may be used as resources for DNA barcoding and molecular marker development for further genetic diversity analyses and molecular marker-assisted breeding. The whole cp genome phylogenetic tree revealed that the 51 samples representing 7 *Neocinnamomum* taxa were divided into six branches. Among them, *N. lecomtei* was the most primitive, basal taxon, and *N. delavayi* was found to be most closely related to *N. fargesii*. These results deepen our understanding of the *Neocinnamomum* cp genome and provide the basis for subsequent taxonomic identification, phylogenetic evolution, and population genetics studies of *Neocinnamomum* species.

## Data availability statement

The 50 new sequencing data presented in the study are deposited in the NCBI (https://www.ncbi.nlm.nih.gov/nuccore), accession numbers OR085909- OR085920, and OR095860- OR095897.

## Author contributions

PX and YS designed the research study. YS and WX contributed materials. ZC annotated and analyzed the genomes, wrote the manuscript. LY isolated DNA, assembled the genomes. YX, QL, HZ and YT helped ZC to analyze the data. All authors contributed to the article and approved the submitted version.
