# Supplementary material for: Comparative and phylogenetic analysis of complete chloroplast genomes from seven Neocinnamomum taxa (Lauraceae)
Source: Front Plant Sci. 2023 Jul 7;14:1205051. doi: 10.3389/fpls.2023.1205051 (PMC10362447; doi:10.3389/fpls.2023.1205051)
Supplement: Supplementary file 1 [file Table_1.docx]

**Table S1** Information of *Neocinnamomum* samples

| **No** | **Taxon** | **Sequence No.** | **Sampling Area** | **Sample No.** | **Tacq** |
| --- | --- | --- | --- | --- | --- |
| 1 | *N. delavayi* | 6068 | Yunnan, China | 37073 | 2020/8/31 |
| 2 | *N. delavayi* | 6083 | Guangxi, China | 37088 | 2020/9/7 |
| 3 | *N. delavayi* | 7790 | Wuhan, China | 36746 | 2019/12/6 |
| 4 | *N. delavayi* | 9763 | Wuhan, China | 35377 | 2017/9/4 |
| 5 | *N. delavayi* | 5832 | Yunnan, China | YangQE2905 | 2015/5/6 |
| 6 | *N. lecomtei* | 57830 | Hainan, China | 36768 | 2019/12/23 |
| 7 | *N. lecomtei* | 5800 | Hainan, China | 12CS5706 | 2015/5/6 |
| 8 | *N. lecomtei* | 57820 | Hainan, China | 36767 | 2019/12/23 |
| 9 | *N. lecomtei* | 57821 | Hainan, China | 36768 | 2019/12/23 |
| 10 | *N. lecomtei* | 7753 | Hainan, China | XY25 | 2016/12/6 |
| 11 | *N. lecomtei* | 9528 | Hainan, China | 34485 | 2017/3/9 |
| 12 | *N. lecomtei* | 7562 | Guangdong, China | 34507 | 2017/3/9 |
| 13 | *N. lecomtei* | 7796 | Hainan, China | SY25 | 2019/9/20 |
| 14 | *N. fargesii* | 9100 | Chongqing, China | 34404 | 2017/2/20 |
| 15 | *N. fargesii* | 9101 | Chongqing, China | 34386 | 2017/2/20 |
| 16 | *N. fargesii* | 9494 | Chongqing, China | 34386 | 2017/2/20 |
| 17 | *N. fargesii* | 9850 | Chongqing, China | 34404 | 2017/2/21 |
| 18 | *N. mekongense* | 7683 | Chongqing, China | 36705 | 2019/10/31 |
| 19 | *N. mekongense* | 7777 | Yunnan, China | 34484 | 2017/03/09 |
| 20 | *N. mekongense* | 7778 | Yunnan, China | 34909 | 2017/5/8 |
| 21 | *N. mekongense* | 7782 | Yunnan, China | 34907 | 2017/5/9 |
| 22 | *N. mekongense* | 7781 | Yunnan, China | 34538 | 2017/3/27 |
| 23 | *N. mekongense* | 6057 | Yunnan, China | 37047 | 2020/7/27 |
| 24 | *N. caudatum* var. *macrocarpum* | 9634 | Wuhan, China | D097 | 2017/11/17 |
| 25 | *N. caudatum* var. *macrocarpum* | 9635 | Wuhan, China | D098 | 2017/11/17 |
| 26 | *N.sp* | 7685 | Yunnan, China | 36737 | 2019/11/1 |
| 27 | *N.sp* | 9581 | Yunnan, China | 33249 | 2019/10/3 |
| 28 | *N. caudatum* | 7779 | Yunnan, China | 34908 | 2017/05/08 |
| 29 | *N. caudatum* | 7783 | Yunnan, China | 34904 | 2017/5/8 |
| 30 | *N. caudatum* | 57831 | Yunnan, China | 36779 | 2020/4/11 |
| 31 | *N. caudatum* | 5801 | Yunnan, China | 13CS5913 | 2015/5/6 |
| 32 | *N. caudatum* | 9247 | Laos | L0158 | 2018/3/25 |
| 33 | *N. caudatum* | 6166 | Yunnan, China | 37205 | 2020/12/1 |
| 34 | *N. caudatum* | 9987 | Yunnan, China | 35677 | 2018/10/7 |
| 35 | *N. caudatum* | 57832 | Yunnan, China | 36801 | 2020/4/11 |
| 36 | *N. caudatum* | 57881 | Yunnan, China | 36930 | 2020/5/22 |
| 37 | *N. caudatum* | 9065 | Yunnan, China | ST86 | 2018/7/24 |
| 38 | *N. caudatum* | RL01 | Yunnan, China | - | - |
| 39 | *N. caudatum* | 6104 | Yunnan, China | 37109 | 2020/9/10 |
| 40 | *N. caudatum* | 7751 | Myanmar | M4994 | 2018/11/20 |
| 41 | *N. caudatum* | 57874 | Yunnan, China | 36812 | 2020/3/30 |
| 42 | *N. caudatum* | 9657 | Yunnan, China | 34906 | 2017/5/8 |
| 43 | *N. caudatum* | 9562 | Yunnan, China | 33652 | 2020/4/11 |
| 44 | *N. caudatum* | 57885 | Yunnan, China | 36933 | 2020/5/20 |
| 45 | *N. caudatum* | 57880 | Yunnan, China | 36926 | 2020/5/20 |
| 46 | *N. caudatum* | 57833 | Yunnan, China | 36835 | 2020/4/8 |
| 47 | *N. caudatum* | 9558 | Yunnan, China | 36779 | 2020/03/24 |
| 48 | *N. caudatum* | 7713 | Yunnan, China | 36658 | 2019/9/29 |
| 49 | *N. caudatum* | 7714 | Yunnan, China | 36655 | 2019/9/29 |
| 50 | *N. caudatum* | 5833 | Yunnan, China | 13CS7131 | 2015/5/6 |
| 51 | *N. caudatum* | 7754 | Yunnan, China | AK007 | 2019/12/23 |

*Note: "-" indicates that the sample information is missing. The data of *N. caudatum* (RL01) was downloaded from NCBI database, accession number: MT621576.
