# Supplementary material for: Comparative and phylogenetic analysis of complete chloroplast genomes from seven Neocinnamomum taxa (Lauraceae)
Source: Front Plant Sci. 2023 Jul 7;14:1205051. doi: 10.3389/fpls.2023.1205051 (PMC10362447; doi:10.3389/fpls.2023.1205051)
Supplement: Supplementary file 2 [file Table_2.doc]

**Table S2** Chloroplast genome characteristics of 7 *Neocinnamomum* taxa

| **Taxon** | **Serial number** | **Size (bp)** | | | | **Gene number** | | | | **GC content (%)** | | | |
| --- | --- | --- | --- | --- | --- | --- | --- | --- | --- | --- | --- | --- | --- |
|  |  | **LSC** | **SSC** | **IR** | **Total** | **CDS** | **tRNA** | **rRNA** | **Total** | **LSC** | **SSC** | **IR** | **Mean** |
| *N. delavayi* | 5832 | 91,876 | 18,419 | 20,271 | 150,837 | 81 | 36 | 8 | 125 | 37.4 | 33.3 | 44.6 | 38.8 |
| *N. delavayi* | 6068 | 91,886 | 18,442 | 20,257 | 150,842 | 81 | 36 | 8 | 125 | 37.4 | 33.3 | 44.6 | 38.8 |
| *N. delavayi* | 6083 | 91,872 | 18,419 | 20,271 | 150,833 | 81 | 36 | 8 | 125 | 37.4 | 33.3 | 44.6 | 38.8 |
| *N. delavayi* | 7790 | 91,882 | 18,420 | 20,270 | 150,842 | 81 | 36 | 8 | 125 | 37.4 | 33.3 | 44.6 | 38.8 |
| *N. delavayi* | 9763 | 91,871 | 18,419 | 20,271 | 150,832 | 81 | 36 | 8 | 125 | 37.4 | 33.3 | 44.6 | 38.8 |
| *N. mekongense* | 6057 | 91,850 | 18,372 | 20,267 | 150,756 | 81 | 36 | 8 | 125 | 37.4 | 33.3 | 44.6 | 38.8 |
| *N. mekongense* | 7683 | 91,889 | 18,447 | 20,257 | 150,850 | 81 | 36 | 8 | 125 | 37.4 | 33.2 | 44.6 | 38.8 |
| *N. mekongense* | 7777 | 91,854 | 18,376 | 20,262 | 150,754 | 81 | 36 | 8 | 125 | 37.4 | 33.3 | 44.6 | 38.9 |
| *N. mekongense* | 7778 | 91,853 | 18,376 | 20,262 | 150,753 | 81 | 36 | 8 | 125 | 37.4 | 33.3 | 44.6 | 38.9 |
| *N. mekongense* | 7781 | 91,854 | 18,376 | 20,262 | 150,754 | 81 | 36 | 8 | 125 | 37.4 | 33.3 | 44.6 | 38.9 |
| *N. mekongense* | 7782 | 91,850 | 18,372 | 20,267 | 150,756 | 81 | 36 | 8 | 125 | 37.4 | 33.3 | 44.6 | 38.8 |
| *N. fargesii* | 9100 | 91,888 | 18,422 | 20,266 | 150,842 | 81 | 36 | 8 | 125 | 37.4 | 33.2 | 44.5 | 38.8 |
| *N. fargesii* | 9101 | 91,885 | 18,422 | 20,266 | 150,839 | 81 | 36 | 8 | 125 | 37.4 | 33.2 | 44.5 | 38.8 |
| *N. fargesii* | 9494 | 91,885 | 18,422 | 20,266 | 150,839 | 81 | 36 | 8 | 125 | 37.4 | 33.2 | 44.5 | 38.8 |
| *N. fargesii* | 9850 | 91,880 | 18,422 | 20,266 | 150,834 | 81 | 36 | 8 | 125 | 37.4 | 33.2 | 44.5 | 38.8 |
| *N.sp* | 7685 | 91,902 | 18,412 | 20,257 | 150,828 | 81 | 36 | 8 | 125 | 37.4 | 33.2 | 44.5 | 38.8 |
| *N.sp* | 9581 | 91,906 | 18,412 | 20,257 | 150,832 | 81 | 36 | 8 | 125 | 37.4 | 33.2 | 44.5 | 38.8 |
| *N. caudatum* var. *macrocarpum* | 9634 | 92,003 | 18,428 | 20,262 | 150,955 | 81 | 36 | 8 | 125 | 37.4 | 33.3 | 44.6 | 38.8 |
| *N. caudatum* var. *macrocarpum* | 9635 | 92,004 | 18,428 | 20,262 | 150,956 | 81 | 36 | 8 | 125 | 37.4 | 33.3 | 44.6 | 38.8 |
| *N. lecomtei* | 5800 | 92,006 | 18,101 | 20,424 | 150,955 | 81 | 36 | 8 | 125 | 37.5 | 33.4 | 44.6 | 38.9 |
| *N. lecomtei* | 7562 | 91,987 | 18,096 | 20,425 | 150,933 | 81 | 36 | 8 | 125 | 37.5 | 33.3 | 44.6 | 38.9 |
| *N. lecomtei* | 7753 | 91,932 | 18,096 | 20,425 | 150,878 | 81 | 36 | 8 | 125 | 37.5 | 33.3 | 44.6 | 38.9 |
| *N. lecomtei* | 7796 | 91,987 | 18,096 | 20,425 | 150,933 | 81 | 36 | 8 | 125 | 37.5 | 33.3 | 44.6 | 38.9 |
| *N. lecomtei* | 9528 | 91,987 | 18,096 | 20,425 | 150,933 | 81 | 36 | 8 | 125 | 37.5 | 33.3 | 44.6 | 38.9 |
| *N. lecomtei* | 57820 | 91,995 | 18,096 | 20,425 | 150,941 | 81 | 36 | 8 | 125 | 37.5 | 33.3 | 44.5 | 38.8 |
| *N. lecomtei* | 57821 | 91,996 | 18,096 | 20,425 | 150,942 | 81 | 36 | 8 | 125 | 37.5 | 33.3 | 44.6 | 38.9 |
| *N. lecomtei* | 57830 | 91,996 | 18,096 | 20,425 | 150,942 | 81 | 36 | 8 | 125 | 37.5 | 33.3 | 44.6 | 38.9 |
| *N. caudatum* | 5801 | 91,884 | 18,445 | 20,262 | 150,853 | 81 | 36 | 8 | 125 | 37.4 | 33.2 | 44.6 | 38.8 |
| *N. caudatum* | 5833 | 91,892 | 18,457 | 20,262 | 150,873 | 81 | 36 | 8 | 125 | 37.4 | 33.2 | 44.6 | 38.8 |
| *N. caudatum* | 6104 | 91,892 | 18,457 | 20,262 | 150,873 | 81 | 36 | 8 | 125 | 37.4 | 33.2 | 44.6 | 38.8 |
| *N. caudatum* | 6166 | 91,877 | 18,457 | 20,262 | 150,858 | 81 | 36 | 8 | 125 | 37.4 | 33.2 | 44.6 | 38.8 |
| *N. caudatum* | 7713 | 91,891 | 18,457 | 20,262 | 150,872 | 81 | 36 | 8 | 125 | 37.4 | 33.2 | 44.6 | 38.8 |
| *N. caudatum* | 7714 | 91,890 | 18,457 | 20,262 | 150,871 | 81 | 36 | 8 | 125 | 37.4 | 33.2 | 44.6 | 38.8 |
| *N. caudatum* | 7751 | 91,958 | 18,400 | 20,258 | 150,874 | 81 | 36 | 8 | 125 | 37.4 | 33.3 | 44.6 | 38.8 |
| *N. caudatum* | 7754 | 91,874 | 18,457 | 20,262 | 150,855 | 81 | 36 | 8 | 125 | 37.4 | 33.2 | 44.6 | 38.8 |
| *N. caudatum* | 7779 | 91,892 | 18,457 | 20,262 | 150,873 | 81 | 36 | 8 | 125 | 37.4 | 33.2 | 44.6 | 38.8 |
| *N. caudatum* | 7783 | 91,892 | 18,457 | 20,262 | 150,873 | 81 | 36 | 8 | 125 | 37.4 | 33.2 | 44.6 | 38.8 |
| *N. caudatum* | 9065 | 91,884 | 18,445 | 20,262 | 150,853 | 81 | 36 | 8 | 125 | 37.4 | 33.2 | 44.6 | 38.8 |
| *N. caudatum* | 9247 | 91,895 | 18,457 | 20,262 | 150,876 | 81 | 36 | 8 | 125 | 37.4 | 33.2 | 44.6 | 38.8 |
| *N. caudatum* | 9558 | 91,892 | 18,457 | 20,262 | 150,873 | 81 | 36 | 8 | 125 | 37.4 | 33.2 | 44.6 | 38.8 |
| *N. caudatum* | 9562 | 91,892 | 18,457 | 20,262 | 150,873 | 81 | 36 | 8 | 125 | 37.4 | 33.2 | 44.6 | 38.8 |
| *N. caudatum* | 9657 | 91,892 | 18,457 | 20,262 | 150,873 | 81 | 36 | 8 | 125 | 37.4 | 33.2 | 44.6 | 38.8 |
| *N. caudatum* | 9987 | 91,889 | 18,457 | 20,262 | 150,870 | 81 | 36 | 8 | 125 | 37.4 | 33.2 | 44.6 | 38.8 |
| *N. caudatum* | 57831 | 91,892 | 18,457 | 20,262 | 150,873 | 81 | 36 | 8 | 125 | 37.4 | 33.2 | 44.6 | 38.8 |
| *N. caudatum* | 57832 | 91,894 | 18,457 | 20,262 | 150,875 | 81 | 36 | 8 | 125 | 37.4 | 33.2 | 44.6 | 38.8 |
| *N. caudatum* | 57833 | 91,892 | 18,457 | 20,262 | 150,873 | 81 | 36 | 8 | 125 | 37.4 | 33.2 | 44.6 | 38.8 |
| *N. caudatum* | 57874 | 91,902 | 18,457 | 20,262 | 150,883 | 81 | 36 | 8 | 125 | 37.4 | 33.2 | 44.6 | 38.8 |
| *N. caudatum* | 57880 | 91,892 | 18,457 | 20,262 | 150,873 | 81 | 36 | 8 | 125 | 37.4 | 33.2 | 44.6 | 38.8 |
| *N. caudatum* | 57881 | 91,885 | 18,445 | 20,262 | 150,854 | 81 | 36 | 8 | 125 | 37.4 | 33.2 | 44.6 | 38.8 |
| *N. caudatum* | 57885 | 91,892 | 18,457 | 20,262 | 150,873 | 81 | 36 | 8 | 125 | 37.4 | 33.2 | 44.6 | 38.8 |
| *N. caudatum* | RL01 | 91,879 | 18,447 | 20,257 | 150,840 | 81 | 36 | 8 | 125 | 37.4 | 33.2 | 44.6 | 38.8 |
